# ProtGO: universal protein function prediction utilizing multi-modal gene ontology knowledge

**DOI:** 10.1093/bioinformatics/btaf390

**Published:** 2025-07-09

**Authors:** Boyan Wang, Yangliao Geng, Xingyi Cheng, Bo Chen, Zhilei Bei, Wei Wang, Jie Tang, Le Song

**Affiliations:** School of Intelligence Science and Technology, Nanjing University, Suzhou, Jiangsu 215163, China; Computer Science and Technology, Tsinghua University, Beijing 100084, China; Key Laboratory of Big Data & Artificial Intelligence in Transportation (Ministry of Education), School of Computer Science and Technology, Beijing Jiaotong University, Beijing 100044, China; BioMap Research, Beijing 100190, China; Computer Science and Technology, Tsinghua University, Beijing 100084, China; Computer Science and Technology, Tsinghua University, Beijing 100084, China; Computer Science and Technology, Tsinghua University, Beijing 100084, China; College of Computer Science, Nankai University, Tianjin 300350, China; Computer Science and Technology, Tsinghua University, Beijing 100084, China; GenBio AI, MBZUAI, Abu Dhabi, United Arab Emirates

## Abstract

**Motivation:**

As one of the recalcitrant challenges in life sciences and biomedicine, protein function prediction suffers from a deluge of AI-designed proteins, particularly having to face multi-modal information in the era of big data. Importing the high-throughput neural-network-based prediction framework to replace the low-throughput biological experiments, a universal multi-modal method is straightforward in addressing the growing gap between known sequences and predicting functions.

**Results:**

To bridge the gap, we propose ProtGO, a three-step framework for predicting protein function, which leverages the credible Gene Ontology (GO) knowledge base and integrates four common modalities. Specifically, we first introduce frontier pre-trained protein language models (PLMs) for representation learning of mainstay functional protein sequences. For the remaining multi-modal data, we design a text alignment module for explainable text descriptions, a taxonomy encoding module for species-specific taxonomy, and a GO graph embedding module for biological GO relations. Each module is independent and adaptive for the referenced modalities. By harnessing these four knowledge representations, ProtGO maximizes the potential of GO resources, enhancing the performance of vanilla PLMs and biological language models (LMs) in downstream GO prediction tasks. Extensive experiments demonstrate that ProtGO significantly advances the abilities of state-of-the-art PLMs to predict protein functions: approximately 8% to 27% increase in the maximum F1 measure (*F*max) compared to base models. These comprehensive studies confirm ProtGO’s capability to deliver outstanding performance in protein function prediction by utilizing a rich blend of functional and evolutionary knowledge.

**Availability and implementation:**

Our source code and all the data are available at https://github.com/sunyatawang/ProtGO.

## 1 Introduction

Protein function prediction is crucial for understanding biological processes, accelerating drug discovery, and elucidating disease mechanisms (Kreitz *et al.* 2023, Kulmanov *et al.* 2024). Traditional functional analyses are based on biology with long-term development cycles, thus needing sufficient human and financial support. Moreover, low-throughput biological experiments have become increasingly inadequate in the era of big data, severely limiting progress in proteomics research (Abramson *et al.* 2024, Zhou *et al.* 2024). The rapid emergence of novel nucleic acid sequences generated by programmable generative models further exacerbates the challenge (Ingraham *et al.* 2023). Thus, there is an urgent need to establish efficient, high-throughput computational methods for transferring functional annotations from well-characterized proteins to newly identified sequences. Predicting Gene Ontology (GO) terms is a promising strategy for discerning biological roles and understanding the life process (Flamholz *et al.* 2024).

Established in 1998, the Gene Ontology (GO) knowledge base (Zhou *et al.* 2019) serves as an authoritative bioinformatics resource for describing gene product function, providing the world’s most comprehensive framework for systematically annotating across diverse species. Compared to other protein function annotation tasks (such as fluorescence prediction), GO-based function prediction provides a universal, comprehensive, and fundamental framework for exploring the multi-faceted nature of proteins (Xu *et al.* 2023), making GO terms the gold standard in protein function annotation.

**Figure 1.**
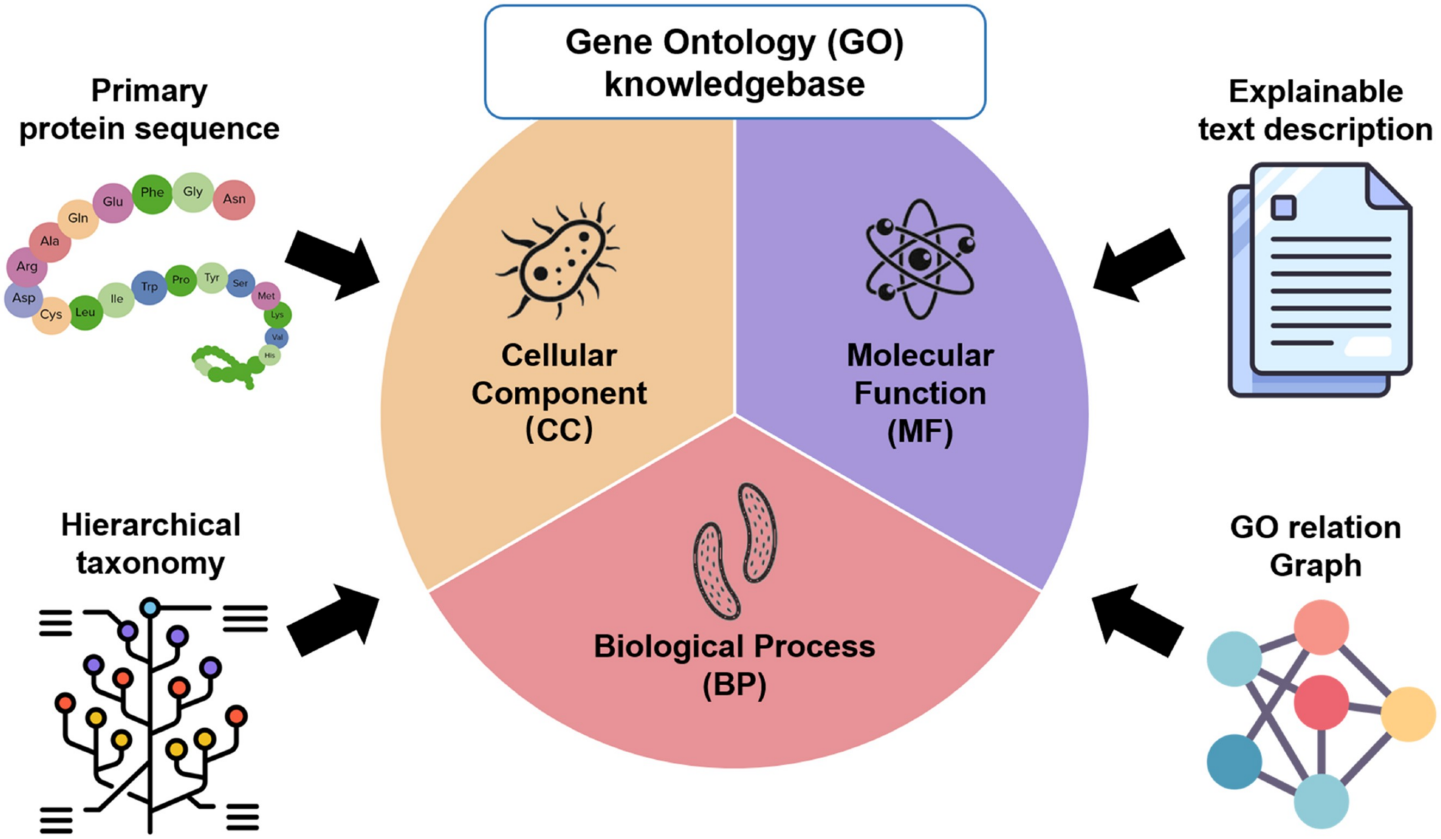
Four modalities in the GO knowledge base: protein sequence, text description, hierarchical taxonomy, and relation graph.

Specifically, the GO resource categorizes biological functions into three distinct subontologies: biological process (BP), molecular function (MF), and cellular component (CC) (Ashburner *et al.* 2000). As shown in Fig. 1, most open-source GO terms in each subontology come with four common feature modalities for prediction, i.e., four types of knowledge (Aleksander *et al.* 2023). These modalities include: (i) the primary protein sequence, a linear amino acid sequence fundamentally governing biological properties; (ii) textual descriptions, offering interpretable biological and biomedical context; (iii) hierarchical taxonomy, specifying species-level classification; and (iv) the GO relation graph, explicitly encoding relationships among GO terms. An open challenge is how to efficiently predict the specific functions of the AI-designed protein while extracting valuable knowledge from referred modalities.

Recent works for GO prediction depend mainly on the primary protein sequence using pre-trained protein language models (PLMs) (Lin *et al.* 2023). Owing to the development of large-scale language models (LMs) (Thirunavukarasu *et al.* 2023), LMs can handle thousands of questions parallelly and efficiently. Inheriting the framework of LMs, PLMs have the natural advantage of conducting large-scale sequence representation simultaneously to address the exponentially increasing demands in biological sequence analysis. Moreover, PLMs effectively capture evolutionary patterns and intrinsic sequence features without relying heavily on extensive multiple sequence alignments (Baek *et al.* 2021, Jumper *et al.* 2021). Meanwhile, state-of-the-art GO prediction methods typically enhance PLMs by incorporating only one additional GO-associated modality, such as textual labels (Boadu and Cheng 2024) or relational graphs (Tian *et al.* 2023). Recent multi-modal frameworks combining PLMs with classical GO prediction methods have demonstrated notable performance improvements (Zhang *et al.* 2022).

However, a major limitation remains: existing GO prediction models tend to utilize a limited subset of available GO-related knowledge. Most methods combine the primary protein sequence with just one additional modality, disregarding other modalities that are often considered insufficiently specific for accurate annotations (Xu *et al.* 2023). This raises a critical research question: Are all GO-related feature modalities beneficial for protein function prediction? If so, how can we effectively integrate these varied modalities alongside the primary protein sequence to achieve accurate and robust predictions?

To comprehensively investigate the value of each GO modality, we propose **ProtGO**, a universal computational framework designed to integrate multiple modalities available within the GO knowledge base for precise protein function annotation. Understanding the relationship between amino acid sequences and protein functions remains a fundamental challenge (Bileschi *et al.* 2022) with significant scientific and translational implications. ProtGO leverages state-of-the-art PLMs to encode primary sequence representations and systematically integrates additional modalities through a knowledge-enhanced pipeline consisting of three distinct modules. The textual alignment module employs self-attention mechanisms (Vaswani *et al.* 2017) to incorporate interpretable textual descriptions. The taxonomy encoding module uses label encoding techniques (Yadav 2019) to capture species-specific information. Lastly, the GO graph embedding module leverages graph neural networks (GNNs) (Peng *et al.* 2022) to exploit relational knowledge embedded in the GO term hierarchy. With these strategic integrations, ProtGO effectively combines multiple GO modalities to yield accurate multi-label GO term predictions.

Practically, ProtGO presents a modular and flexible pipeline that can dynamically incorporate relevant submodules depending on the available modalities for each protein sequence prediction task. The main contributions of our work are summarized as follows:

1. We propose ProtGO, a universal pipeline that endows comprehensive multi-modal knowledge for protein function prediction. ProtGO effectively captures functional and evolutionary information to improve the GO prediction and validate the effect of each modal knowledge.
2. Powered by text descriptions, hierarchical taxonomy, GO relation graphs, and the foundational primary protein sequences, ProtGO is adaptive and lightweight for any scalable PLMs and biological LMs over the high-throughout knowledge-enhanced framework.
3. Extensive experiments show that ProtGO-enhanced models outperform the vanilla PLMs with 8% to 27% improvements in maximum F1 measure (*F*max), which also advances the state-of-the-art GO prediction methods by a large margin based on the specialized submodule design.

## 2 Preliminaries

As the world’s most comprehensive resource of protein functions, GO describes our knowledge of the biological domain concerning three aspects: BP, MF, and CC. BP subontology encompasses biological programs accomplished by multiple molecular activities; MF subontology describes functional activities that occur at the molecular level; CC subontology pertains to the localization of gene products, relative to cellular compartments and structures, occupied by a macromolecular machine (Ashburner *et al.* 2000). Three aspects of GO terms are designed to be graph-based, accommodating GO relevant to both prokaryotic and eukaryotic organisms, including those that are single-celled or multicellular, across various levels of organisms (Gligorijević *et al.* 2021). Hence, protein function prediction based on GO terms is an expert multi-label classification system considering multi-modal information and complex data structures in the corresponding BP, MF, and CC domains.

From the perspective of multi-label classification (Tarekegn *et al.* 2021), the GO prediction task involves a learning challenge using multi-modal knowledge-enhanced embeddings. Given a dataset of *n* protein samples with GO terms $S=\{(X_{i},Y_{i})|1\leq i\leq n\}$, where $X_{i}$ represents a mixed feature embedding and $Y_{i}\in {\left\{0,1\right\}}^{\gamma \times 1}$ is a binary label vector composed of γ GO terms. The group of all sample feature embeddings that combine multiple modalities is referred to as the feature space X. Similarly, the collection of all label vectors, encapsulating the inter-label relations, constitutes the label space Y. The objective of the protein prediction problem is to derive the function *F* from the feature space to the label space, i.e. $X\overset{F}{\to }Y$.

## 3 Materials and methods

**Figure 2.**
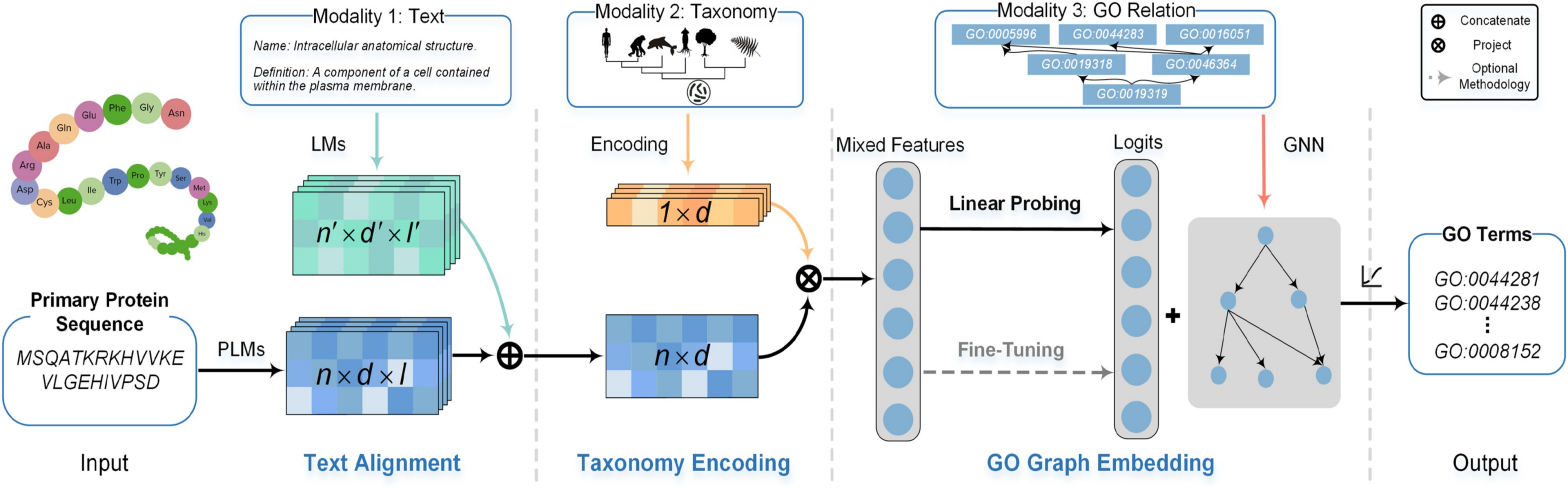
Illustration of ProtGO framework. Abbreviations: LM, language model; PLM, protein language model. Owing to three modules, ProtGO merges three additional types of knowledge: explainable text description, hierarchical species-specific taxonomy, and GO relation graph into the mainstay PLMs representation for the GO prediction. The three-step framework is flexible to satisfy various real-world samples with actual additional modalities.

As shown in Fig. 2, the core work of three-step ProtGO is learning multi-modal knowledge to enhance the primary protein sequence representations based on PLMs. The framework incorporates two methodologies for multi-label GO classification, i.e. linear probing with MLP (He *et al.* 2022) (default) and fine-tuning with LoRA (Hu *et al.* 2021) (optional). Naive feature alignment often suffers in poor performance due to the absence of credible correlation with other modalities (Shen *et al.* 2023). In this section, we aim to integrate text description, hierarchical taxonomy, and GO relation graph in a lightweight and adaptive manner to form the mixed functional representation. Each alignment step is model-agnostic, ensuring compatibility with any PLM. Moreover, ProtGO adopts one specialized strategy for one modality as an independent submodule, implying that the proposed ProtGO is modularizable. Each submodule is flexible for referred knowledge and aims to help ProtGO obtain further performance bonuses. Based on the ablation study, we verify the effect of each module in Section 4.3.

### 3.1 Explainable text alignment module

For many proteins, explainable text and hierarchical taxonomy are two common knowledge in the feature space of the GO resource. Text descriptions directly relate to the protein function aligning with protein sequences, such as the name and definition of GO terms, as shown in Fig. 2. Therefore, ProtGO first merges the textual representation with the protein sequence representation using PLMs. Owing to any biological and biomedical pre-trained LMs (Gu *et al.* 2022, Yasunaga *et al.* 2022), informative representations can be extracted from the text descriptions about protein function (Van Remmen *et al.* 2021). However, the gap between the two modalities and the irrelevant text representation hinders the alignment. Consequently, ProtGO primarily focuses on selecting and incorporating the relevant text representation corresponding to the sequence representation provided by PLMs.

To measure the relation between the final-layer embedding from LMs for text representation $X^{\mathrm{text}}$ and the final-layer embedding from PLMs for protein sequence representation $X^{\mathrm{seq}}$, ProtGO unifies the dimension of the two embeddings based on the mainstay $X^{\mathrm{seq}}$. Specifically, the core protein sequence representation $X^{\mathrm{seq}}=\{x_{1}^{\mathrm{seq}},\ldots ,x_{n}^{\mathrm{seq}}\}$ consists of *n* biological embeddings, where $x_{i}^{\mathrm{seq}}\in {\mathbb{R}}^{d\times l}$ indicates *l* residues with the embedding dimension *d*. The text representation $X^{\mathrm{text}}=\{x_{1}^{\mathrm{text}},\ldots ,x_{n^{\prime }}^{\mathrm{text}}\}$ has $n^{\prime }$ textual embeddings, each embedding $x_{i}^{\mathrm{text}}\in {\mathbb{R}}^{d^{\prime }\times l^{\prime }}$ indicates $l^{\prime }$ tokens with the embedding dimension $d^{\prime }$. Through a linear layer, each textual embedding is projected to match the dimension *d* of the biological sequence embedding. With the cosine similarity:


(1) $$\text{cos}\;(x_{i}^{\mathrm{text}},x_{j}^{\mathrm{seq}})=\frac{1}{l}\sum_{k=0}^{l}\frac{x_{ik}^{\mathrm{text}}\cdot x_{jk}^{\mathrm{seq}}}{\left||x_{ik}^{\mathrm{text}}||\times ||x_{jk}^{\mathrm{seq}}|\right|}$$

where *k* is the number of vectors. ProtGO selects the relevant text representation (i.e. $\text{cos}\;(x_{i}^{\mathrm{text}},x_{j}^{\mathrm{seq}})>0$) to concatenate with the sequence representation.

In order to further fuse the two modalities, we introduce a self-attention mechanism for aligning with the relevant text representation. Given the order of protein sequences and the relevant textual tokens, we conduct sine and cosine functions PE() as the positional encoding for both knowledge embeddings. Each attention head $\mathrm{Attention}(X^{\mathrm{cat}})$ is based on the concatenation $X^{\mathrm{cat}}=PE(X^{\mathrm{seq}})⊕PE(M(X^{\mathrm{text}}))$ between the protein sequence representation and the filtered text representation $M(X^{\mathrm{text}})$, where *M* signifies the function to project and select the relevant textual embeddings:


(2) $$\text{Attention}(X^{\mathrm{cat}})=\text{softmax}(\frac{X^{\mathrm{cat}}{\left(X^{\mathrm{cat}}\right)}^{T}}{\sqrt{d}})X^{\mathrm{cat}}$$

Owing to the classical position-wise feed-forward network and softmax function, ProtGO obtains the explainable multi-modal learning embeddings at the amino acid level by the text-alignment module. Compared with traditional multi-modal learning, our text alignment module aligns the distinct textual properties with sequences using the low-cost semantic transformer instead of the high-cost pre-training setup in the supervised classification task.

In this paper, we employ eight parallel attention heads. The dot-product attention is adopted for its higher efficiency than traditional additive attention (Vaswani *et al.* 2017), so it can quickly fuse sequence and text embeddings through optimized alignment coding. Moreover, ProtGO splits the linear *l* amino acid sequences and $l^{\prime }$ tokens into feature fragments, each with a length of 512. The split facilitates the alignment of long protein sequences or contexts by breaking them into manageable segments. The text alignments module is a specialized bridge to accelerate the information transfer from the semantic textual properties into the mainstay functional sequence representation.

### 3.2 Hierarchical taxonomy encoding module

As one of the crucial elements of bioscience, most proteins in the GO knowledge base possess a complete taxonomy that describes their species within a hierarchical structure, showcasing co-evolutionary relationships among organisms to pinpoint accurate functions. The principal ranks in modern taxonomy include domain, kingdom, phylum, class, order, family, genus, and species (Wheeler 2004). Considering applicable to prokaryotes and eukaryotes for GO terms, ProtGO focuses primarily on the leaf node of the hierarchical taxonomy, i.e. the specific species or type genus of the protein. With all the possible species (You *et al.* 2021) in the encoding module, ProtGO realizes the multi-species as additional features to cluster functional protein representations, especially for sparse species.

For the alignment results of sequences and text features at the amino acid level, we compute the mean value across the dimensions of $l+l^{\prime }$ to achieve the feature alignment $X^{\mathrm{pro}}$ at the protein level. Through compact label encoding LE() of taxonomy:


(3) $$X^{\mathrm{mix}}=LE(X^{\mathrm{tax}})w_{\mathrm{tax}}+X^{\mathrm{pro}}$$

We project the taxonomy information $X^{\mathrm{tax}}$ with the weight $w_{\mathrm{tax}}$ to yield the mixed feature embeddings $X^{\mathrm{mix}}\in {\mathbb{R}}^{n\times d}$. We conduct a linear layer to search for the best value of weight $w_{\mathrm{tax}}$. For three subontologies, most ProtGO-enhanced PLMs obtain the optimal performances when the value of weight $w_{\mathrm{tax}}$ is close to 0.1. Hence, we set the weight $w_{\mathrm{tax}}$ of the projection of taxonomy embeddings as 0.1 to merge the species-specific knowledge. Given the species-agnostic character of most GO vocabulary, label encoding is a lightweight and robust technique for hierarchical multi-species encoder.

### 3.3 Prediction via GO relation graph module

Following this partitioning, ProtGO prunes the low frequent GO terms to avoid the long-tail effect (Zhang and Zhou 2014) and reduce the complexity in advance. Based on the dataset distribution and experimental results, the GO prediction obtains optimal performance when the pruning GO frequency nearly reaches 0.015% of the whole samples. We provide details about the GO frequency in Section 4.8. With the above preprocessing GO label space, each GO subgraph can be characterized by a normalized adjacency matrix $\hat{A}$. Utilizing this, we first apply a two-layer perceptron to process the mixed features $X^{\mathrm{mix}}$, then incorporate a graph convolution operation to leverage the information from the GO subgraph for generating the final prediction result:


(4) $$F(X^{\mathrm{mix}})=\sigma_{A}\left(\hat{A}(\sigma_{1}(X^{\mathrm{mix}}W_{1}+b_{1})W_{2}+b_{2})W_{A}\right)$$

where $W_{1}$, $W_{2}$, $W_{A}$, $b_{1}$, and $b_{2}$ represent learnable parameters, while $\sigma_{A}$ and $\sigma_{1}$ are non-linear activations implemented as sigmoid and ReLU functions (Han and Moraga 1995), respectively. As the final learning modality, the succinct GO relation graph module reinforces the mixed feature by adding DAG structure knowledge in the label space, thus achieving the goal of high-throughout function prediction.

## 4 Results and discussion

### 4.1 Experimental setup

#### 4.1.1 Experimental dataset

We evaluate the proposed ProtGO on the 5th Critical Assessment of protein Function Annotation (CAFA5) dataset, which contains open-source and high-quality protein sequences with corresponding GO terms. CAFA5 protein function prediction dataset has 142 246 tagged protein sequences with 31 466 GO terms, referring to 3156 species in the taxonomy. Under the principle that all the GO terms must be present for training, we split the tagged samples randomly into 128 020/14 225 samples as the train/test set. To validate the robustness of the original dataset and the splitting strategy, more analyses about the dataset are in Section 4.7.

#### 4.1.2 Baselines

To validate the generality of our ProtGO to any PLM, we test its performance using most existing PLMs with various scales. We apply ProtGO with three advanced pre-trained PLMs, i.e. Ankh-Large (parameters: 2B) (Elnaggar *et al.* 2023), ESM-2 series (parameters: 150M, 650M, 3B, and 15B) (Lin *et al.* 2023), and xTrimoPGLM (parameters: 101B) (Chen *et al.* 2025). For protein function prediction via GO terms, we analyze the experimental performance using linear probing with MLP and fine-tuning with LoRA methodologies. For a fair comparison, we employ BioMedBERT-abs (Gu *et al.* 2022) as the default biological LMs for the text representation with a dropout rate of 0.3. More experimental results of the other LMs are shown in Section 4.6. Furthermore, we compare with the performant GO prediction models, e.g., OntoProtein (Zhang *et al.* 2022), DeepFRI (Gligorijević *et al.* 2021), ProtST (Xu *et al.* 2023), DeepGOGAT (Kulmanov *et al.* 2024), PROTGOAT (Chua et al. 2024), and InterLabelGO+ (Liu *et al.* 2024).

To compare the baselines fairly, all the baselines are trained or tested on the same training/test subsets. We also follow the optimal setting in the corresponding reference. For our ProtGO pipeline, we conduct Adam optimizer with default setting ($\beta_{1}$ = 0.9, $\beta_{2}$ = 0.999, and $\varepsilon =10^{-9}$). For Ankh, ESM2-150M, and ESM2-650M, we employ ProtGO with the initial learning rate of $2\times 10^{-4}$, then is decreased by a factor of 0.6 every five epochs. The rest is the initial learning rate of $4\times 10^{-5}$, which is decreased by 0.5 every five epochs. For linear probing with MLP, we train all the models over 30 epochs using the BCE loss with a batch size of 16. For fine-tuning with LoRA, we conduct over 25 epochs with r=8 and α=16 using BCE loss, utilizing a batch size of 1. The dimension of the hidden layer in the two-layer perceptron is 2×d based on the embedding size *d* of the pre-trained PLMs.

Experimental results are shown in protein-centric evaluation measure *F*max (Clark and Radivojac 2013) and traditional multi-label classification metrics, e.g. example-based F1 score (F1), area under the precision-recall curve (AUPR), and Matthews correlation coefficient (MCC) (Zhang and Zhou 2014, Giles *et al.* 2017).

### 4.2 Protein function prediction performance via GO terms

**Figure 3.**
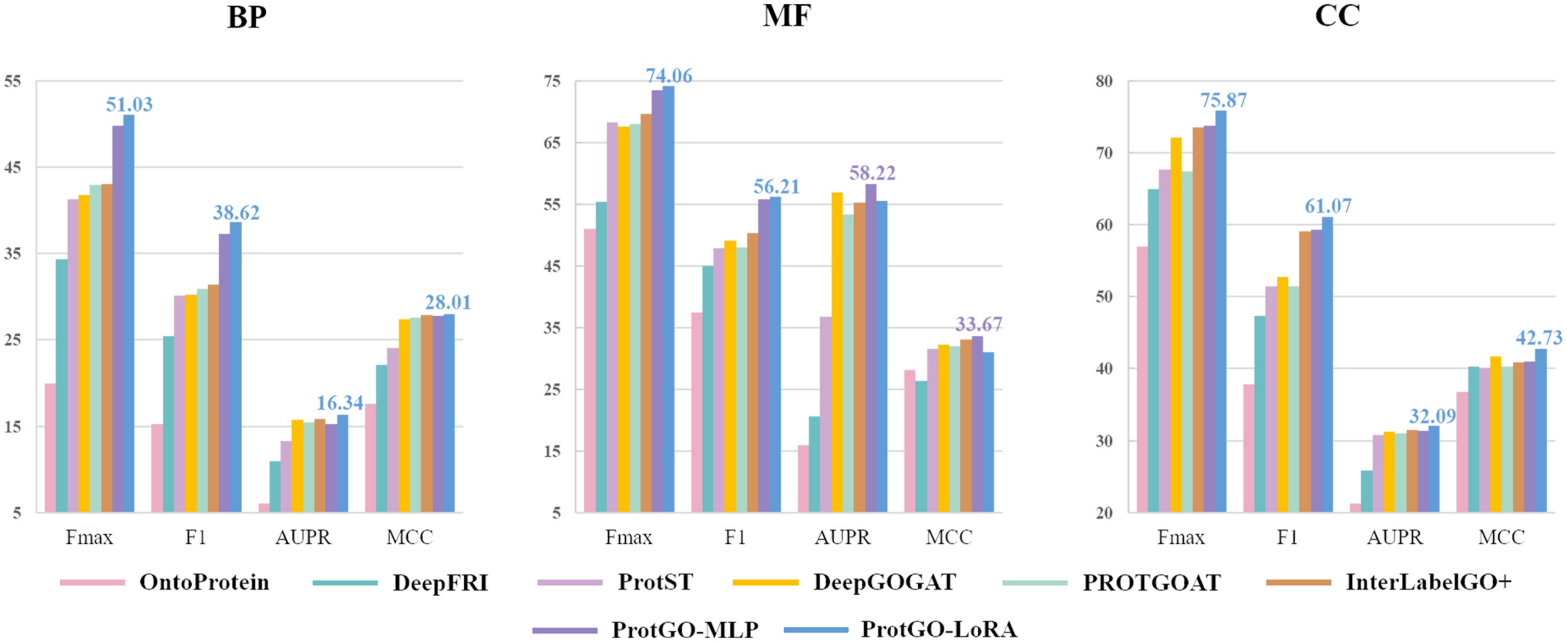
Bar charts of GO prediction in Fmax, F1, AUPR, and MCC (%). Abbreviations: F1, example-based F1 score; ProtGO-MLP, ProtGO-enhanced linear probing with MLP; ProtGO-LoRA, ProtGO-enhanced fine-tuning with LoRA. The three subfigures show three subontologies. Optimal performances on four metrics are shown with a specific index in the subfigure. To fairly compare with baselines, we conduct ESM2-650M as the base PLM for all the models. Each bar chart has eight methods, and each method plots a curve with a unique color.

Table 1 represents the GO prediction results of CAFA5 in *F*max and example-based F1 score using frontier PLMs. As shown in Fig. 3, we compare ProtGO using two refining methodologies to the downstream function classification task with specialized GO prediction baselines. Without additional explanations, the default methodology of the GO prediction methods is linear probing with MLP. Based on the benchmark results in various PLMs and comparison with GO prediction models, we highlight the observations as follows:

**Table 1.** Experimental results with PLMs on CAFA5 protein function dataset (%).^a^

Method	BP	MF	CC	Method	BP	MF	CC	Method	BP	MF	CC
Measure: F **max**											
Ankh	17.44	50.56	59.68	ESM150M	20.86	55.81	61.15	ESM650M	24.10	60.27	62.87
Ankh-LoRA	–	–	–	ESM150M-LoRA	31.47	62.60	69.12	ESM650M-LoRA	29.23	62.30	67.17
**ProtGO-Ankh**	**42.10**	**67.56**	**68.99**	**ProtGO-ESM150M**	**47.51**	**71.84**	**73.04**	**ProtGO-ESM650M**	**49.76**	**73.09**	**73.92**
ESM3B	28.70	63.80	66.24	ESM15B	33.57	65.91	67.12	PGLM	34.32	69.90	70.39
ESM3B-LoRA	31.91	64.36	68.85	ESM15B-LoRA	–	–	–	PGLM-LoRA	34.43	74.07	74.47
**ProtGO-ESM3B**	**48.71**	**72.86**	**74.51**	**ProtGO-ESM15B**	**50.78**	**73.63**	**74.67**	**ProtGO-PGLM**	**55.08**	**78.81**	**79.65**
Measure: **F1**											
Ankh	16.34	36.45	39.97	ESM150M	19.91	40.41	41.12	ESM650M	21.72	43.06	43.15
Ankh-LoRA	–	–	–	ESM150M-LoRA	28.71	48.66	48.70	ESM650M-LoRA	26.06	43.26	48.17
**ProtGO-Ankh**	**29.62**	**48.92**	**50.37**	**ProtGO-ESM150M**	**34.46**	**53.80**	**56.36**	**ProtGO-ESM650M**	**37.31**	**56.22**	**59.31**
ESM3B	25.67	46.23	46.14	ESM15B	29.11	47.57	49.51	PGLM	30.95	47.99	51.56
ESM3B-LoRA	26.59	45.71	49.84	ESM15B-LoRA	–	–	–	PGLM-LoRA	31.23	50.12	54.89
**ProtGO-ESM3B**	**38.01**	**57.03**	**60.04**	**ProtGO-ESM15B**	**38.47**	**57.41**	**59.73**	**ProtGO-PGLM**	**39.25**	**57.32**	**60.29**

a Abbreviations: PGLM, xTrimoPGLM; F1, example-based F1 score. The local best results for each base model are highlighted in **bold**.

ProtGO is an outstanding knowledge-enhanced pipeline via GO terms for protein function prediction, even better than the fine-tuned results of the base PLM. For various PLMs in Table 1, ProtGO-extended models outperform the linear-probing and fine-tuning results of the original ones. Overall, ProtGO-enhanced models outperform the vanilla PLMs with around 8%-27% improvements. For the F1 score, ProtGO achieves similar significant performances based on the original ones with an 8%–16% increase in results. Given the imbalance in GO prediction, F1 scores are usually lower than the corresponding *F*max measures to reflect the prediction results per sample. Moreover, compared to the fine-tuning results by LoRA, linear probing results with ProtGO still keep the local best performance per base PLM. ProtGO is more professional for protein function prediction via GOs than the traditional downstream refining methodology.

Our ProtGO pipeline is adaptive and lightweight for any PLMs with various scales and frameworks, while improvements in GO prediction by ProtGO transcend the scale benefits of the model. In Table 1, ProtGO-enhanced ESM150M models outperform the valina ESM15B in terms of both *F*max and F1, even defeats PGLM-LoRA in F1 score. The overall improvements using PLMs based on the proposed ProtGO transcend the scale of base PLM, reflecting that ProtGO utilizes PLMs with multiple modalities. On the other hand, the improvements validate that the GO prediction is a comprehensive and complex protein function task, which requires a professional pipeline to handle the multiple knowledge in the GO resource. ProtGO-enhanced xTrimoPGLM is the best-performing method with the average *F*max of 71.18% and average F1 of 52.29% in accord with its scale and training data. ProtGO inherits and maintains the advantage of PLMs in the GO terms annotation task, which boosts their performance with a consistent rise.

Models based on PLM outperform the traditional models for protein function prediction, validating that the protein sequence modality makes the largest contribution to GO prediction among various knowledge. Under the scale of 650 million, OntoProtein and DeepFRI cannot defeat the performance of the PLM-extended model in four metrics, as shown in Fig. 3. Even with additional knowledge, OntoProtein and DeepFRI only match the performance of the vanilla ESM3B in Table 1. The benchmark performance gaps between traditional protein function models and PLM-based ones show the remarkable effectiveness of PLMs, especially in learning biological function information from protein sequences.

ProtGO-LoRA predicts better but makes the prediction pipeline computationally more expensive than ProGO-MLP since ProtGO-MLP has fully harnessed the potential of the PLMs. As shown in Fig. 3, the fine-tuning technique by the LoRA strategy over the ProtGO did not yield evident enhancements, specifically ≤1.5% average improvements in both *F*max and F1. In contrast, ProtGO-LoRA’s enhanced prediction capability decreases in AUPR and MCC for MF subontology (ProtGO-MLP defeats ProtGO-LoRA). Besides, the running time of supervised fine-tuning is usually more than twice that of linear probing for the same base PLM. ProtGO breaks the principle that LoRA delivers more stable performance enhancements than MLP. The findings underscore the superiority of ProtGO, which leverages multi-modal knowledge, over the LoRA fine-tuning methodology.

The more comprehensive modalities the multi-modal learning involves, the stronger prediction of the protein function performs. Most baselines only import two types of knowledge for multi-modal learning, such as OntoProtein, DeepFRI, and ProtST. Given the multi-modal learning during the pre-training process, pre-trained ProtST has consistently outperformed OntoProtein and DeepFRI. Based on two modalities, DeepGOGAT cooperates with protein-protein interactions to form three modalities. Similarly, PROTGOAT incorporates protein sequence with taxonomy and textual function, and the proposed ProtGO unifies four modalities in the GO knowledge base. Thus, PROTGOAT ranks third, DeepGOGAT ranks fourth, and our ProtGO-series ranks first regarding the protein function classifier based on the ESM650M in most metrics. Hence, merging as many available modalities as possible of each protein is a better choice for GO annotation.

### 4.3 Effect of each module in ProtGO

**Figure 4.**
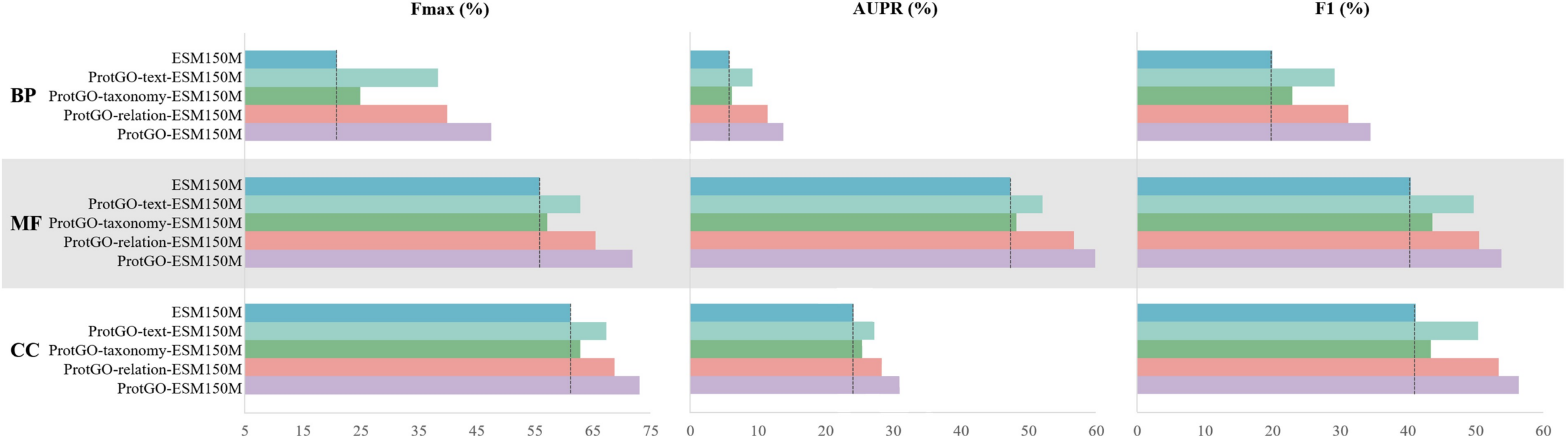
Ablation study of each independent submodule of ProtGO based on ESM150M in terms of *F*max, AUPR, and F1. Abbreviations: ProtGO-text, ProtGO only with text alignment module; ProtGO-taxonomy, ProtGO only with taxonomy encoding module; ProtGO-relation, ProtGO only with GO relation graph module. The dashed line indicates the base ESM150M performance.

Compared with the vanilla ESM150M, each component of ProtGO boosts the performance with a relative modality in Fig. 4. In detail, the comprehensive ProtGO has the best performance, secondly to ProtGO-relation, thirdly to ProtGO-text, fourthly to ProtGO-taxonomy, and lastly to the original ESM150M. Among these modalities, the GO relation with the DAG structure, i.e., ProtGO-relation-ESM150M, improves the benchmark performances by the most significant margin. This improvement shows that exploiting the relationships between GO terms is valid knowledge for multi-label classification. ProtGO-taxonomy-ESM150M provides the minimum gains, mainly caused by the species-agnostic character of the GO vocabulary.

In advance, we randomly combine two modalities to form three joint-submodule frameworks to test the fusion and cooperation capability of ProtGO in Table 2. Compared with Fig. 4, the prediction performances with two additional modal knowledge have further gains than the ones with only one extra modality. Especially in terms of the knowledge of hierarchical taxonomy, it is a splendid supplemental modality for text and relation. GO prediction achieves the desired protein function prediction with more valuable modalities involved. The improvements with arbitrary submodules validate that the four kinds of knowledge in the GO resource deserve full utilization for the protein function prediction. Hence, by adding independent modality using the specialized design of the ProtGO submodule, this framework successfully enhances primary protein sequence representation step by step under the purpose of the high-throughout prediction pipeline.

**Table 2.** Ablation study with two joint submodules of ProtGO based on ESM150M (%).^a^

Method	Metrics	BP	MF	CC
ESM150M		20.86	55.81	61.15
ProtGO-*text&taxon*		40.52	64.77	69.34
ProtGO-*taxon&relation*	*F*max	41.92	67.40	70.73
ProtGO-*text&relation*		42.79	68.23	71.34
ProtGO-ESM150M		**47.51**	**71.84**	**73.04**
ESM150M		5.74	47.32	24.07
ProtGO-*text&taxon*		10.15	53.29	27.34
ProtGO-*taxon&relation*	AUPR	12.84	57.92	29.01
ProtGO-*text&relation*		12.99	57.20	29.66
ProtGO-ESM150M		**13.75**	**59.83**	**30.96**
ESM150M		19.91	40.41	41.12
ProtGO-*text&taxon*		30.13	52.75	51.69
ProtGO-*taxon&relation*	F1	32.62	52.93	54.62
ProtGO-*text&relation*		34.18	53.45	55.02
ProtGO-ESM150M		**34.46**	**53.80**	**56.36**

a Abbreviations: ProtGO-text&taxon, joint submodules with text alignment and taxonomy encoding of ProtGO; ProtGO-taxon&relation, joint submodules with taxonomy encoding and GO relation graph of ProtGO; ProtGO-text&relation, joint submodules with text alignment and GO relation graph of ProtGO. We use bold and underline to denote the **best** and the second performances for each metric.

### 4.4 Mapping of GO prediction to sites on protein structures

**Figure 5.**
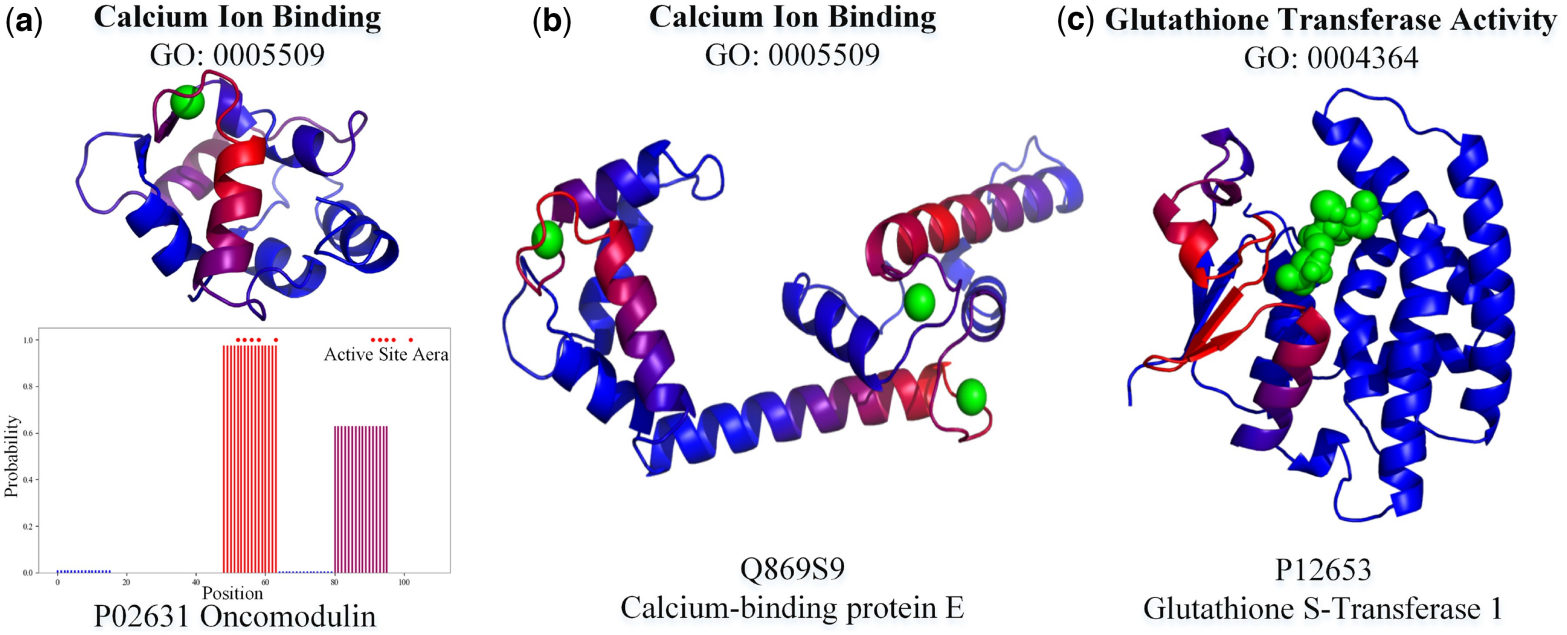
Automatic mapping of function prediction to sites on protein structures. We apply a gradient color scheme to map a specific protein function to corresponding residues, with more relevant residues in red and less relevant residues in blue. The corresponding probability legend is on the right.

Most nodes in the GO relation graph have a specialized protein function with a clear function-to-structure relationship. MF subontology contains a large number of terms arising from the protein structure. GO terms in MF subontology can figure out proteins with similar sequences but have different functions, depending on the referred active sites and the organisms in which they are a part (Kulmanov *et al.* 2024). Hence, a robust GO prediction model can provide the corresponding protein structure by local functional residue fragments via GO terms (Gligorijević *et al.* 2021). To validate the GO prediction results by ProtGO, we employ our GO terms annotation to map the binding sites on the structures of ligands and proteins from the Protein Data Bank, thus reflecting our benchmark results at the level of local amino acid sequences. The docking of ligands to proteins is then calculated by AutoDock Vina (Morris *et al.* 2009) and visualized by PyMOL (DeLano *et al.* 2002). We split the complete protein sequences into several fragments with an average length of 16, as shown in the chart of Fig 5a. Each fragment references binding sites retrieved from the BioLip (Yang *et al.* 2013) and UniProt (Consortium, 2019) databases based on the MF subgraph. For each fragment, we compute the potential probabilities of the crucial residues corresponding to specific GO terms, i.e. function sites of protein structure.

In Fig. 5, we propose three cases to verify GO annotation results by ProtGO-ESM3B and ProtGO-PGLM in the micro perspective. In Fig. 5a, ProtGO-ESM3B successfully predicts the GO: 0005509 as one of the credible tags for Oncomodulin (protein ID: P02631). GO: 0005509 indicates that Oncomodulin is a parvalbumin-family protein binding to a calcium ion (Ca2+). ProtGO-ESM3B automatically maps one among two potential binding areas by the protein function prediction of the corresponding protein fragments. Figure 5b is the other sample for the function of calcium ion binding. Among three possible calcium-binding areas, the ProtGO-enhanced model successfully detects all the sites without co-factors or site specificity information during the learning process.

Figure 5c corresponds to glutathione transferase activity by ProtGO-PGLM. This case shows that ProtGO is available not only for metal-ion binding but also for active sites of an organic. Glutathione transferase activity is critical for cellular detoxification against xenobiotics and noxious compounds against oxidative stress. Hence, the protein with this function is an essential index in cancer detection and chemotherapeutic drug resistance. Based on ProtGO-enhanced models, the significant performing samples via GO terms are sensitive to known site-specific mechanisms or site-specific underpinnings by the function-to-structure relationship.

### 4.5 Protein–Protein interaction via GO prediction results

Especially for CC subontology, most GO terms inherently rely on multiple proteins being present and interacting with each other (Gu *et al.* 2023). Thus, proteins with similar functions, i.e. with same GO terms, are inclined to share similar interaction partners or have physical (direct) associations between the proteins (Zhou *et al.* 2019, Gao *et al.* 2023). Besides, some protein function models have predicted GO by the protein-protein interactions (Wang *et al.* 2022, Kulmanov *et al.* 2024). Hence, we conduct the protein pairs with similar predicted GO terms to verify ProtGO-ESM3B results from the macro perspective. In detail, we keep the predicted samples by ProtGO with above 75% in precision. Among these samples, we further select 578 sample pairs (179 pairs for BP, 216 pairs for MF, and 183 pairs for CC) with similar GO terms (i.e. each pair has ≥70% same GO terms). Due to the sparsity of the GO label space, we only compute the similarity of GO terms based on the positive annotations.

**Figure 6.**
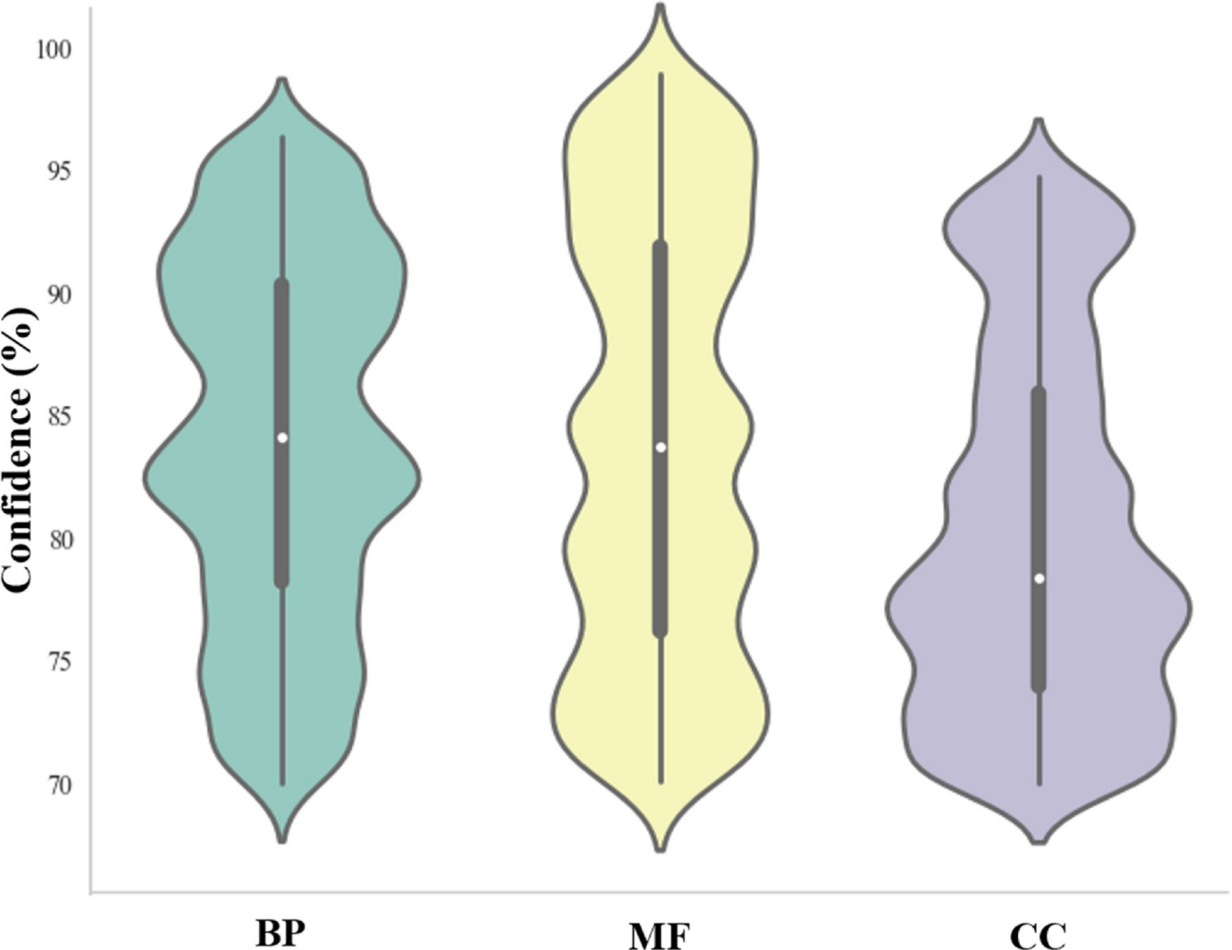
Sample pairing via similar GO prediction results on mapping protein-protein interaction confidence scores (%). The violin plot per subontology depicts the distribution of protein interacting pairs, categorized by similar predicted GO terms within BP, MF, and CC domains. These violin plots mark the median, upper and lower quartiles, and 1.1× interquartile range referred to the confidence scores (≥70%).

In Fig.6, we finally match 403 sample pairs with the corresponding interacting confidence scores based on the STRING database (Szklarczyk *et al.* 2023), which have potential interactions with each other or have the same interaction partner. In STRING, a confidence score above 70% states that an interaction exists, ranging from 0 to 99.9. Specifically, the mapping interacting samples are 141 out of 179 pairs for BP, 153 out of 216 pairs for MF, and 109 out of 183 pairs for CC. The matching rate is approximately 69.72% for the whole reserved pairs, demonstrating that GO terms are valid for exploring protein-protein interactions. The mapping of function-to-interaction relationships further proves the benchmark results trustworthy.

### 4.6 Comparisons of biological LMs

The proposed ProtGO is universal to general PLMs as well as to pre-trained biological and biomedical LMs. Hence, we employ three specialized pre-trained LMs to show the universal merits of the proposed ProtGO: BioMedBERT-abs, BioMedBERT-full (Gu *et al.* 2022), and BioLinkBERT (Yasunaga *et al.* 2022). In Table 3, we present the benchmark performance of ProtGO-ESM150M in terms of *F*max, AUPR, F1, and MCC under the default setup. The gaps among the three LMs are about 1% on four metrics, owing to the similar text representation for limited relevant text descriptions. ProtST (Xu *et al.* 2023) utilizes BioMedBERT-abs as the default LM. In order to make a fair comparison with baselines, ProtGO applies the default BioMedBERT-abs for text representation based on robust performances. Compared with the vanilla ESM150M, ProtGO is beneficial to any LMs, borne out by the significant improvements in the combination of Table 1 and Table 3. Regardless of the choices for frontier PLMs and biological LMs, the proposed ProtGO pipeline provides consistent gains for learning protein sequences with text descriptions.

**Table 3.** Performance comparison of ProtGO-ESM150M with different LMs (%).^a^

Method	Metrics	BP	MF	CC
BioMedBERT-abs		47.51	71.84	73.04
BioMedBERT-full	*F*max	46.74	71.34	72.28
BioLinkBERT		**47.79**	**72.19**	**73.78**
BioMedBERT-abs		**13.75**	59.83	30.96
BioMedBERT-full	AUPR	13.28	59.39	30.29
BioLinkBERT		13.66	**60.20**	**30.99**
BioMedBERT-abs		34.46	53.80	56.36
BioMedBERT-full	F1	34.16	53.78	56.13
BioLinkBERT		**35.17**	**54.62**	**57.01**
BioMedBERT-abs		26.20	30.58	**40.43**
BioMedBERT-full	MCC	26.16	29.96	40.11
BioLinkBERT		**27.17**	**30.73**	40.20

a The best results are highlighted in **bold** for each metric.

### 4.7 Dataset analysis

For multi-label classification, the data distribution affects the experimental results directly due to the sparsity of the label space and the long-tail effect (Zhang and Zhou 2014). As a specialized classifier, GO prediction must analyze the data first to avoid the distinct diversity of sample distribution between the training and test sets. Cross-validation experiment (Geisser, 2017) is a straightforward way to check the influence of data distribution for baselines. Given the inconsistent additional modalities for samples, we focus on the distribution over the primary protein sequences, which is the foundational information for protein function prediction. Besides, we analyze the data distribution computed over the example-based statistics. Hence, we conduct five-fold cross-validation on the base PLMs in example-based F1 scores.

**Figure 7.**
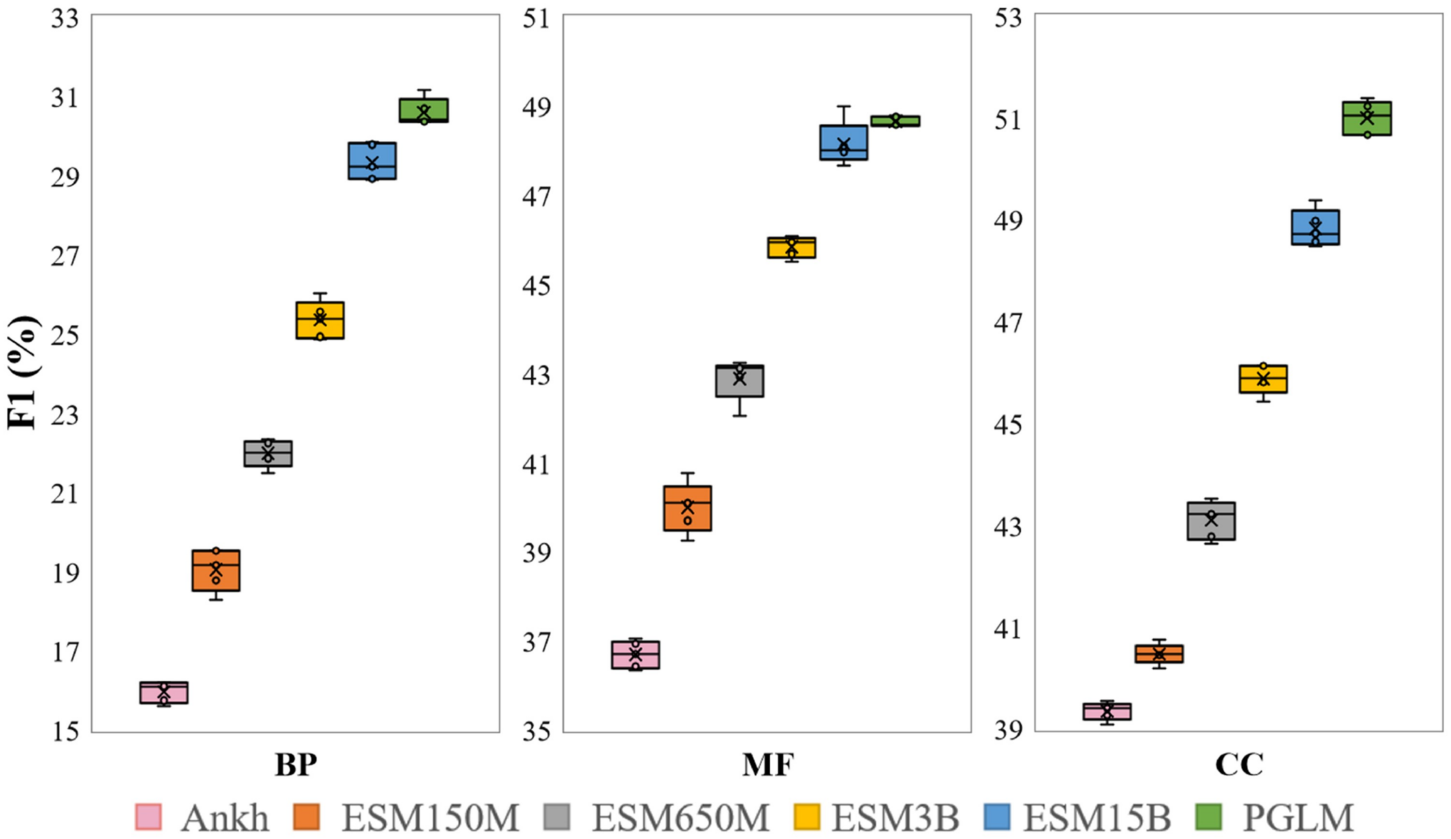
Five-fold cross-validation results on CAFA5 with base PLMs in terms of F1. The box plots show BP, MF, and CC subontology prediction performances, i.e. the minimum, the maximum, the sample median, and the first and third quartiles.

In Fig. 7, we show F1 performances with all the original PLMs to show the robustness and stability of any scalable PLMs. The data distribution of protein sequence representation does not distort the prediction results of GO terms. The variability in terms of F1 between each round is within 1.5%. The largest standard deviation of referred PLMs is controlled to 0.5% in the five-fold experiments. Figure 7 has validated that the CAFA5 dataset is uniformly distributed for the sequence modality. Given the computational resources, we split the tagged samples randomly into 128 020/14 225 samples as the train/test set for GO prediction.

With the growth of the scale, PLMs have better performance in F1 scores based on the efficient sequence representation. Hence, we leverage PLMs as the cornerstone of the proposed ProtGO to handle the mainstay sequence knowledge.

### 4.8 Effect of GO frequency

**Figure 8.**
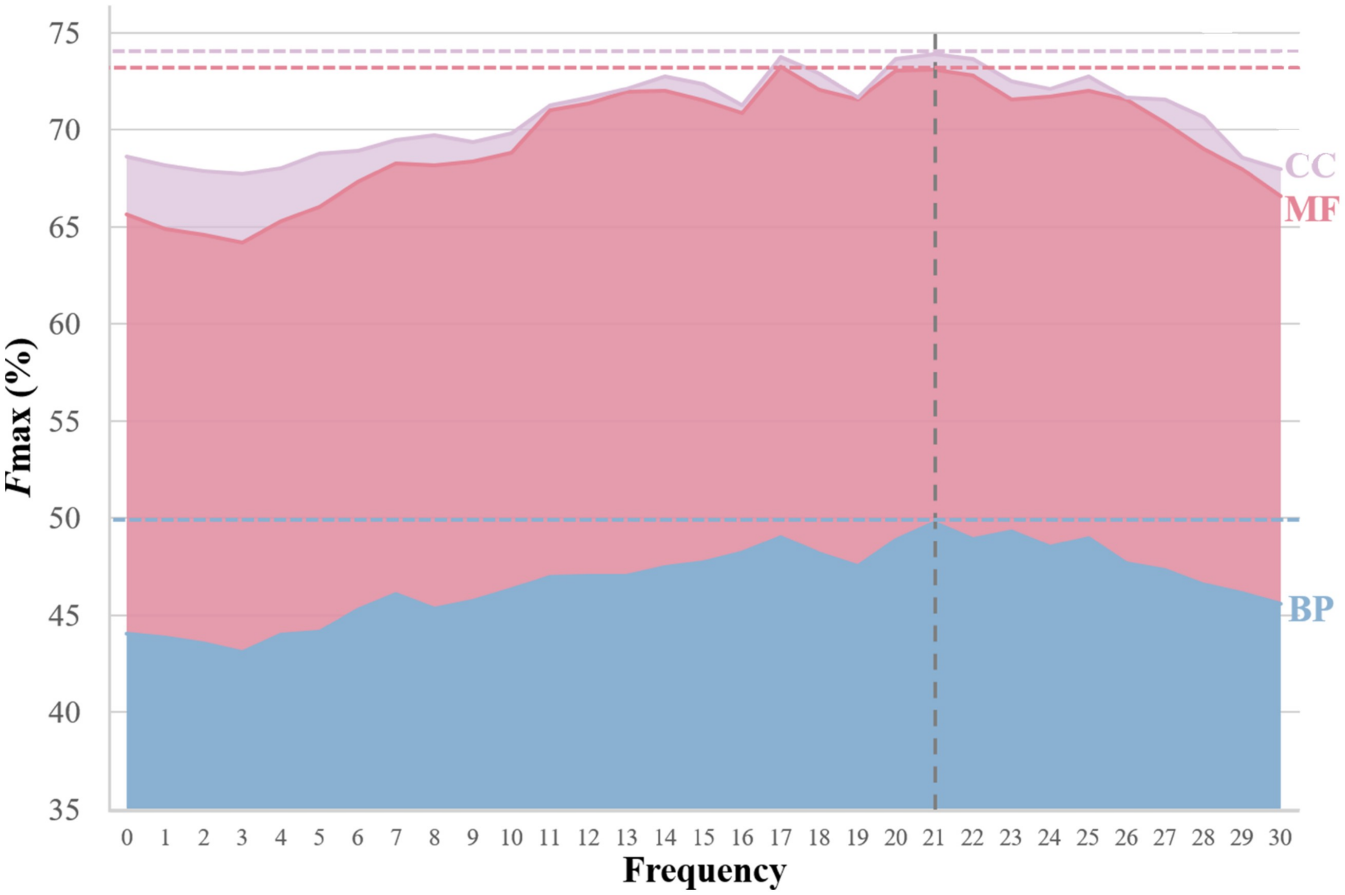
Prediction performance of ProtGO-ESM650M varying with the incremental pruning GO frequencies in terms of *F*max. The dashed line indicates the average optimal results in three subontologies with the corresponding frequency of 21.

To avoid the long-tail effect in the multi-label classification, we handle the GO terms in the label space by pruning the low frequent annotations (Park *et al.* 2024). As shown in Fig. 8, the prediction performance in terms of *F*max forms an approximate concave function with the increasing frequency value. The frequency indicates the occurrence of GO terms in the CAFA5 dataset. The low frequency reflects that few samples are tagged with the corresponding GO terms. While frequency reaches 21, i.e., about 0.015% of the whole samples, ProtGO-ESM650M obtains the optimal performance since we set the default value of pruning frequency as 21. Specifically, the pruning GO terms are 13 641 out of 21 285 for BP, 5740 out of 7224 for MF, and 1932 out of 2957 for CC. The GO prediction reserves 32.27% high-frequent GO terms with a 6.35% average *F*max improvement. Hence, by downsizing a large redundant training GO set into a small high-frequency GO subset, we can reduce the enormous computational costs and improve the prediction performance.

## 5 Conclusion

In this work, we explored the feasibility of integrating diverse multi-modal knowledge with protein sequence representations derived from pre-trained protein language models (PLMs) for enhanced protein function prediction. Leveraging the extensive Gene Ontology (GO) knowledge base, our proposed ProtGO framework systematically incorporates explainable textual descriptions, hierarchical taxonomic information, and GO relational graphs into primary sequence embeddings, creating a comprehensive, knowledge-enriched representation. Each modality incrementally and significantly improved prediction performance. Furthermore, ProtGO is highly flexible and readily adaptable to various PLM architectures as well as biological language models of different scales. Specifically, the ProtGO-enhanced model achieved an average improvement of 14.66% in the maximum F1 measure (*F*max) on the CAFA5 dataset compared to vanilla PLM-based models, surpassing even the fine-tuning performance of larger-scale PLMs. Extensive empirical results and rigorous analyses confirmed our hypothesis that all modalities of GO knowledge significantly contribute to protein function prediction, and demonstrated that the universal ProtGO framework paired with PLMs constitutes a robust, high-throughput multi-modal classifier for GO term annotation.

## Data Availability

The data underlying this article are available in the Github website, at https://github.com/sunyatawang/ProtGO.
